# Paradoxical Acceleration of Dithiothreitol-Induced Aggregation of Insulin in the Presence of a Chaperone

**DOI:** 10.3390/ijms11114556

**Published:** 2010-11-15

**Authors:** Zoya Bumagina, Bella Gurvits, Natalya Artemova, Konstantin Muranov, Boris Kurganov

**Affiliations:** 1 A. N. Bakh Institute of Biochemistry, Russian Academy of Sciences, Leninsky prospect 33, Moscow 119071, Russia; E-Mails: kojolschaucy@mail.ru (Z.B.); ximikk@gmail.com (N.A.); kurganov@inbi.ras.ru (B.K.); 2 Emanuel Institute of Biochemical Physics, Russian Academy of Sciences, Kosygina 4, Moscow 119991, Russia; E-Mail: kmuranov@hotbox.ru

**Keywords:** aggregation, alpha-crystallin, dynamic light scattering, insulin

## Abstract

The kinetics of dithiothreitol (DTT)-induced aggregation of human recombinant insulin and the effect of α-crystallin, a representative of the family of small heat shock proteins, on the aggregation process have been studied using dynamic light scattering technique. Analysis of the distribution of the particles by size measured in the course of aggregation showed that the initial stage of the aggregation process was the stage of formation of the start aggregates with a hydrodynamic radius (*R*_h_) of about 90 nm. When studying the effect of α-crystallin on the rate of DTT-induced aggregation of insulin, it was demonstrated that low concentrations of α-crystallin dramatically accelerated the aggregation process, whereas high concentrations of α-crystallin suppressed insulin aggregation. In the present study, at the molar stoichiometric ratio (insulin:α-crystallin) less than 1:0.5, a pronounced accelerating effect of α-crystallin was observed; whereas a ratio exceeding the value of 1:0.6 caused suppression of insulin aggregation. The mechanisms underlying the dual effect of α-crystallin have been proposed. It is assumed that heterogeneous nucleation occurring on the surface of the α-crystallin particle plays the key role in the paradoxical acceleration of insulin aggregation by α-crystallin that may provide an alternative biologically significant pathway of the aggregation process.

## 1. Introduction

α-Crystallin, a member of the small heat shock protein (Hsp) family, functions as a molecular chaperone by interacting with unfolded or misfolded proteins to prevent their aggregation [1]. It has a broad molecular mass distribution of 300 to 1000 kDa and forms large oligomer assemblies that display great heterogeneity [2].

In a previous paper [3], we investigated the effect of α-crystallin on the kinetics of dithiothreitol (DTT)-induced aggregation of α-lactalbumin using dynamic light scattering (DLS). At all concentrations of α-crystallin studied, the chaperone induced an increase in the duration of the lag period on the dependences of the light scattering intensity on time. However, at relatively low concentrations of α-crystallin, an increase in the initial rate of aggregation registered after the completion of the lag period was observed.

To reveal the molecular mechanisms of the accelerating effect of low concentrations of α-crystallin, we decided to study the kinetics of DTT-induced aggregation of a number of other protein substrates. In the present work we investigated the effect of α-crystallin on the kinetics of DTT-induced aggregation of human recombinant insulin in a wide range of the α-crystallin concentrations. This choice was determined by the following considerations: On one hand, the development of modern concepts of protein folding and aggregation has resulted in the discovery of numerous nonpathogenic proteins and peptides, having the ability to form amyloid-like structures, which can induce conformational diseases [4]. Insulin is especially interesting in this aspect because aggregation of insulin induced by disorder in its native structure can cause numerous pathological states. Studies of the protective role of chaperone-like proteins that prevent insulin aggregation may be important for neuroendocrinology, as many pathogenetic problems of a neurodegenerative nature and several other diseases are associated with dysfunctions of insulin [5].

On the other hand, insulin is widely used as a model substrate in folding studies since it forms various oligomeric forms and supramolecular structures as a result of self-assembly or aggregation [6–8]. The protein hormone insulin is synthesized in the β cells of the pancreas where it is stored as a Zn^2+^-containing hexamer. Its large size, however, prevents its efficient absorption into the blood stream. Hexamers are therefore broken down to dimers and then monomers that are transported efficiently into the blood stream [6]. The circulating biologically active form of insulin is a Zn^2+^-free monomer consisting of two chains linked by two disulfide bridges. Insulin can be induced to aggregation by the reduction of S–S bridges between its A and B chains resulting in their dissociation and formation of amorphous aggregates, but under non-physiological conditions, such as high temperatures (60–70 °C), low pH (1.5–2.0) and relatively high protein concentrations, insulin generates amyloid-like fibrils [9–11].

Therefore, it is becoming increasingly important to study the structural transformations of insulin, especially under conditions that promote more extensive aggregation processes, not only from the point of view of the long-term storage or pharmaceutical use of insulin, but also for the more general insight it may provide into the processes of protein aggregation. In spite of extensive studies [12–21], the mechanisms involved in aggregation of insulin under stress conditions, and in the interaction of α-crystallin with the denatured substrate thereby influencing its aggregation, are far from being clear.

In this paper, a detailed study of the kinetics of DTT-induced aggregation of insulin in the absence and presence of α-crystallin has been performed. The measurements of the light scattering intensity and the size distribution of protein particles formed in the course of aggregation allowed us to develop the mechanisms underlying seemingly paradoxical accelerating effect of low concentrations of α-crystallin.

## 2. Results

### 2.1. Kinetics of Dithiothreitol-Induced Aggregation of Insulin

**Figure 1.**
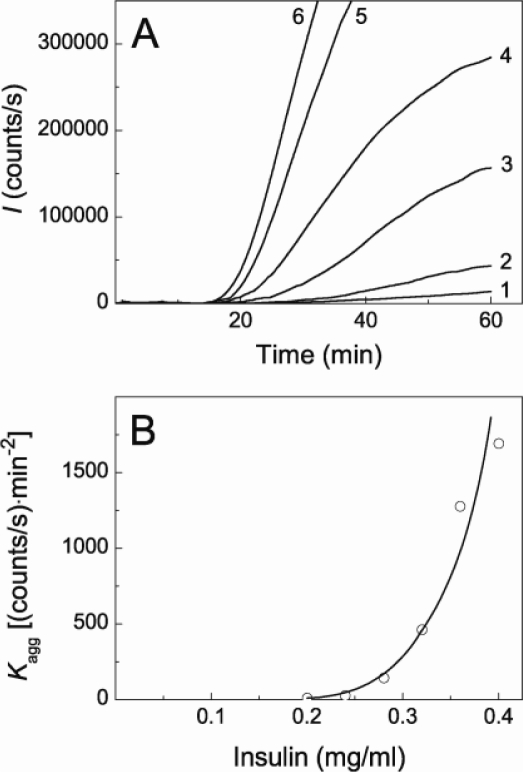
Kinetics of DTT-induced aggregation of insulin. The dependences of the light scattering intensity (*I*) on time obtained at 25 °C in 50 mM sodium phosphate buffer, pH 7.0, containing 0.15 M NaCl and 20 mM DTT (**A**). The concentrations of insulin were as follows: 0.20 (1), 0.24 (2), 0.28 (3), 0.32 (4), 0.36 (5) and 0.40 (6) mg/mL. The dependence of parameter *K*_agg_ on the concentration of insulin (**B**). The points are the experimental data. The solid curve was calculated from Equation (4) (see Experimental Section).

DTT-induced aggregation of insulin was studied by DLS at 25 °C in 50 mM sodium phosphate buffer, pH 7.0, containing 0.15 M NaCl at various concentrations of the protein. Figure 1A demonstrates the increase in the light scattering intensity (*I*) in time accompanying DTT-induced aggregation of insulin. The increase in the concentration of insulin in the interval from 0.2 to 0.4 mg/mL resulted in the increase to the aggregation rate (curves 1–6).

**Figure 2.**
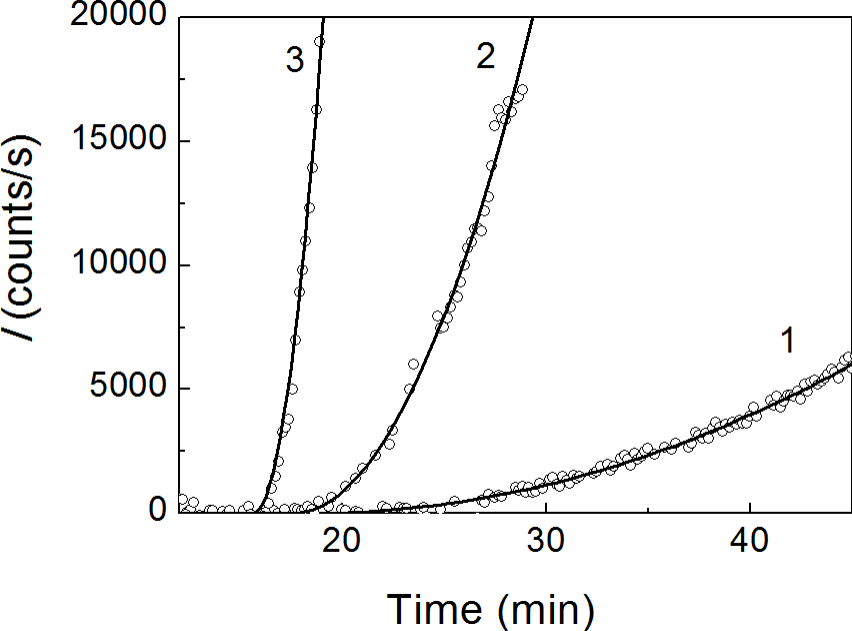
Analysis of the initial parts of the dependences of the light scattering intensity (*I*) on time for DTT-induced aggregation of insulin. The concentrations of insulin were as follows: 0.20 (1), 0.28 (2) and 0.40 (3) mg/mL. The final concentration of DTT is 20 mM. Points are the experimental data. Solid curves were calculated from Equation (1).

A typical feature of the aggregation curves is the appearance of a lag period. To estimate the duration of the lag period, the initial parts of the dependences of *I* on time were analyzed using Equation (1) (see Experimental Section) containing parameters *t*_2_ (the duration of lag period; in other words, the point in time at which the light scattering intensity begins to increase) and parameter *K*_agg_ characterizing the initial rate of aggregation. As an example, Figure 2 shows the fitting of the initial parts of the dependences of *I* on time obtained at concentrations of insulin of 0.20, 0.28 and 0.40 mg/mL.

The values of parameters *t*_2_ and *K*_agg_ calculated at various concentrations of insulin are given in Table 1. The duration of lag period decreases from 18.4 ± 0.5 to 15.6 ± 0.2 min, when the concentration of insulin increases in the interval from 0.2 to 0.4 mg/mL. More significant changes are observed in the *K*_agg_ value. Parameter *K*_agg_, which is a measure of the aggregation rate, increases by a factor of 200 at twofold increase in the concentration of insulin (from 0.2 to 0.4 mg/mL). Figure 1B shows the dependence of parameter *K*_agg_ on the concentration of insulin. The dependence follows the power law in accordance with Equation (4). Parameter *n* was found to be 6.3 ± 0.2.

**Figure 3.**
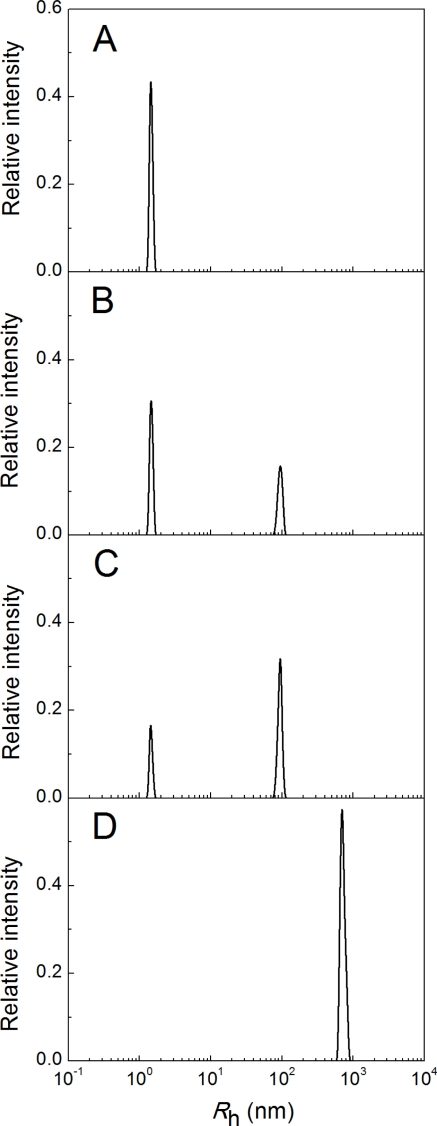
The distribution of the particles by size for insulin. Insulin (0.32 mg/mL) was incubated at 25 °C in the presence of 20 mM DTT for 1 (**A**), 17.2 (**B**), 19.3 (**C**) and 50 (**D**) min.

Consider the time-course of the appearance of the protein aggregates in the solution of insulin after the addition of DTT. Figure 3 shows the insulin distribution of the particles by size (0.32 mg/mL) incubated at 25 °C in the presence of 20 mM DTT. The particle size distributions were recorded at 15 second intervals. Over the first 17 minutes, the single peak with *R*_h_ = 1.3 ± 0.1 nm is registered in the system (Figure 3A; *t* = 1 min). It is evident that this peak corresponds to the non-aggregated form of insulin. At *t* = 17.2 min the start aggregates, with *R*_h_ value higher than 90 nm, appear, and two peaks are registered on the distribution of the particles by size (Figure 3B). The point in time at which the start aggregates appear, was designated as *t*_1_. It should be noted that intermediates between the peaks corresponding to the non-aggregated form of insulin and start aggregates are lacking. Thus, the formation of the start aggregates proceeds on an all-or-none principle.

In the interval from 17.2 to 19.3 min, the position of the peak corresponding to the start aggregates remains unchanged (see, for example, Figure 3C). Since the moment in time at which the start aggregates appear (*t* = 17.2 min), is very close to the precise moment at which the light scattering intensity begins to increase (*t*_2_ = 16.7 min), one can conclude that the increase in the light scattering intensity in the interval from 16.7 to 19.3 min is due to accumulation of the start aggregates in the system; the size of the start aggregates remaining unchanged. At *t* > 19.3 min the size of the start aggregates begins to increase. Figure 3D shows the particle size distribution at *t* = 50 min. The hydrodynamic radius of the protein aggregates reaches a value of about 800 nm.

The analogous character of the changes in the particle size distribution in the solution of insulin after the addition of DTT was observed for other insulin concentrations (0.20, 0.24, 0.28, 0.36 and 0.40 mg/mL). The values of parameters *t*_1_ and *t*_2_ determined at various insulin concentrations are given in Table 1.

**Figure 4.**
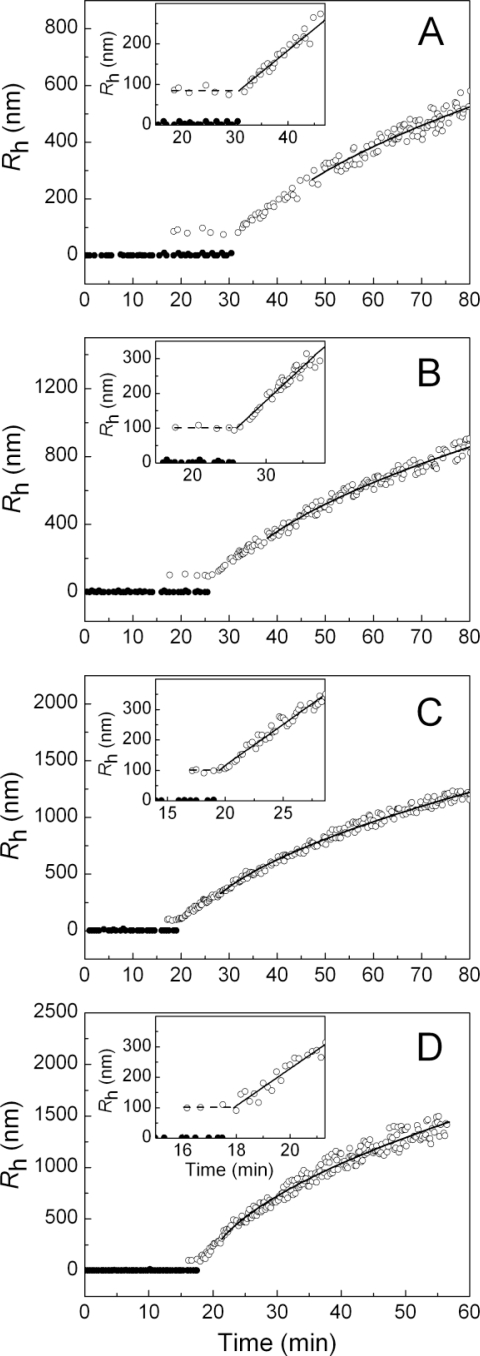
Analysis of the size of the particles in the solution of insulin after the addition of DTT. The dependences of the hydrodynamic radius (*R*_h_) of the non-aggregated form of insulin (solid circles) and protein aggregates (open circles) on time. The concentrations of insulin were as follows: 0.20 (**A**), 0.28 (**B**), 0.32 (**C**) and 0.40 (**D**) mg/mL. The solid curves were calculated from Equation (3). Insets show the dependences of *R*_h_ on time with the expanded ordinate axis. The horizontal dotted lines correspond to the hydrodynamic radius of start aggregates (*R*_h,0_). The solid lines are calculated from Equation (2).

The time-course of the positions of the peaks on the distribution of the particles by size for DTT-induced aggregation of insulin at various concentrations of the protein substrate (in the interval from 0.20 to 0.40 mg/mL) is demonstrated in Figure 4. The solid and open circles correspond to the non-aggregated and aggregated forms of insulin, respectively. The insets show the dependences of *R*_h_ on time with the expanded ordinate axis.

A distinctive feature of the dependences of the hydrodynamic radius of the protein aggregates on time is the constancy of the *R*_h_ values over the definite time interval. This region of the *R*_h_ values corresponds to the start aggregates. The hydrodynamic radius of the start aggregates (*R*_h,0_) remains practically unchanged at variation of the protein concentration (Table 1). The average value of *R*_h,0_ was found to be 94 ± 7 nm.

As can be seen in Figure 4, at a definite moment in time the hydrodynamic radius of the start aggregates begins to increase. This moment in time was designated as *t*_3_. The reason for such an increase in the *R*_h_ value is the sticking of the start aggregates provoked by their accumulation. The initial increase in the *R*_h_ value in time is linear. This allows the value of *t*_3_ to be determined more precisely using Equation (2). The values of parameter *t*_3_ are given in Table 1. The analysis of the values of *t*_2_ and *t*_3_ shows that the time interval (*t*_3_ − *t*_2_), over which the accumulation of the start aggregates occurs without sticking, is shortened by increasing the protein concentration.

Apart from parameter *t*_3_, Equation (2) gives parameter *K*_1_, which characterizes the increase in the *R*_h_ value in time (*K*_1_ = d*R*_h_/d*t*). The values of parameter *K*_1_ are given in Table 1. When the insulin concentration increases from 0.2 to 0.4 mg/mL, a fivefold increase in the *K*_1_ value is observed. The data presented in Figure 3 show that above a definite point in time (*t* > t*), the dependence of *R*_h_ on time follows the power law (see Equation (3). The values of t* and the fractal dimension of the aggregates (*d*_f_) are given in Table 1. The fact that the values of *d*_f_ are close to 1.8 is indicative of the fulfillment of the regime of the diffusion-limited cluster-cluster aggregation.

### 2.2. The Effect of α-Crystallin on the Kinetics of Dithiothreitol-Induced Aggregation of Insulin

**Figure 5.**
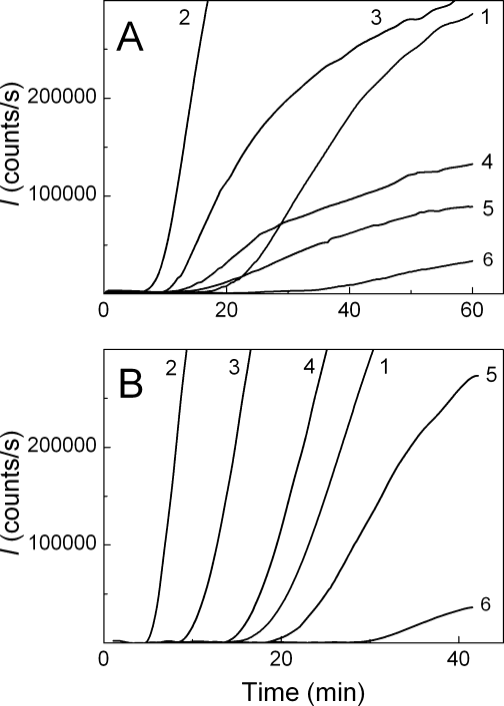
The effect of α-crystallin on the kinetics of DTT-induced aggregation of insulin at the concentrations of 0.32 (**A**) or 0.4 (**B**) mg/mL. The final concentration of DTT was 20 mM. The dependences of the light scattering intensity (*I*) on time at the following concentrations of α-crystallin: 0 (1), 0.08 (2), 0.32 (3), 0.64 (4), 0.8 (5) and 1.20 (6) mg/mL.

To characterize the effect of α-crystallin on the kinetics of DTT-induced aggregation of insulin, we analyzed the time-course of the light scattering intensity (*I*) and the hydrodynamic radius values of the particles formed in the solution of insulin (0.32 and 0.4 mg/mL), after the addition of DTT in the presence of various concentrations of α-crystallin, in the interval from 0.08 to 1.2 mg/mL. Figure 5 shows the kinetic curves of aggregation in the coordinates {*I*; time}. When the concentration of insulin was 0.4 mg/mL, the effect of α-crystallin is clearly visible. The relatively low concentrations of α-crystallin (0.08, 0.32 and 0.64 mg/mL; curves 2, 3 and 4 in Figure 5B, respectively) induce acceleration of insulin aggregation. At higher concentrations of α-crystallin (0.8 and 1.2 mg/mL; curves 5 and 6, respectively) the suppression of insulin aggregation is observed.

At the insulin concentration of 0.4 mg/mL, the decrease in the rate of aggregation with increasing α-crystallin concentration is accompanied by the gradual increase in the duration of lag period. When the insulin concentration was 0.32 mg/mL, the accelerating effect of low concentrations of α-crystallin (0.08, 0.32 and 0.64 mg/mL; curves 2, 3 and 4, respectively) and inhibiting action of high concentrations of α-crystallin (1.2 mg/mL; curve 6 in Figure 5A), are also markedly expressed. However, in general, the character of the effect of α-crystallin on the insulin aggregation is more complicated because the decrease in the aggregation rate with increasing α-crystallin concentration is not accompanied by such a pronounced increase in the duration of lag period as in the case of insulin concentration of 0.4 mg/mL.

**Figure 6.**
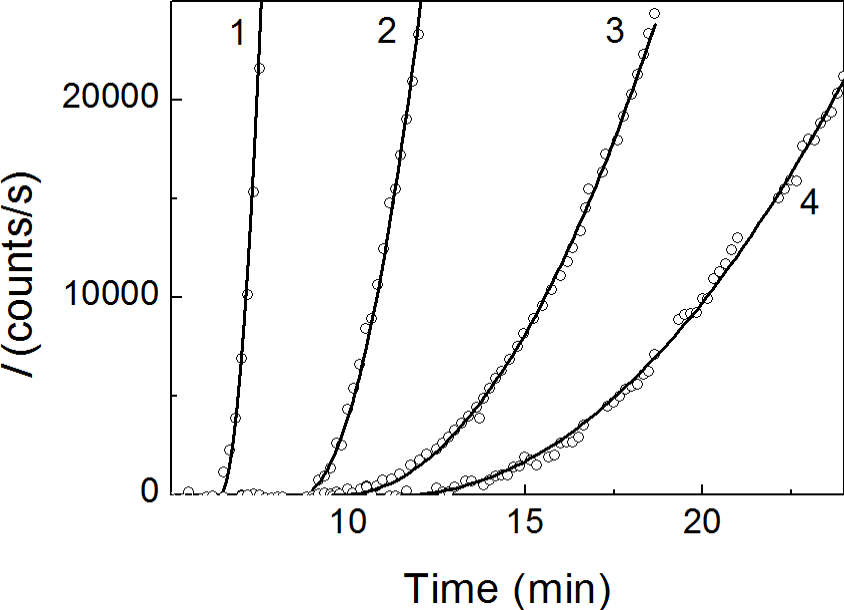
The analysis of the initial parts of the dependences of the light scattering intensity (*I*) on time for DTT-induced aggregation of insulin (0.32 mg/mL). The final concentration of DTT was 20 mM. The concentrations of α-crystallin were as follows: 0.08 (1), 0.16 (2), 0.64 (3) and 0.80 (4) mg/mL. The solid curves are calculated from Equation (1).

It is evident that to characterize the effects caused by different concentrations of α-crystallin, we should understand how α-crystallin influences the duration of lag period, and also understand the initial rate of aggregation. Figure 6 shows that the initial parts of the dependences of the light scattering intensity on time may be satisfactorily described by Equation (1). Thus, the use of this equation enables estimating parameters *t*_2_ (the duration of lag period) and *K*_agg_ (the measure of the initial rate of aggregation). The values of parameters *t*_2_ and *K*_agg_ calculated at the insulin concentrations of 0.32 and 0.4 mg/mL and various concentrations of α-crystallin are given in Table 2. As for the duration of lag period, parameter *t*_2_ becomes lower than the corresponding values measured in the absence of α-crystallin (*t*_2_ = 16.7 or 15.6 min at the insulin concentration of 0.32 or 0.4 mg/mL, respectively). However, at relatively high concentrations of α-crystallin, the values of *t*_2_ exceed the control values. At the molar stoichiometric ratio (insulin:α-crystallin) less than 1:0.5, a pronounced accelerating effect of α-crystallin was observed, whereas at the ratio exceeding the value of 1:0.6 α-crystallin caused suppression of insulin aggregation. These effects could be observed at the concentrations of α-crystallin exceeding 0.64 and 0.8 mg/mL in the experiments with insulin at the concentration of 0.32 and 0.4 mg/mL, respectively (Table 2).

**Figure 7.**
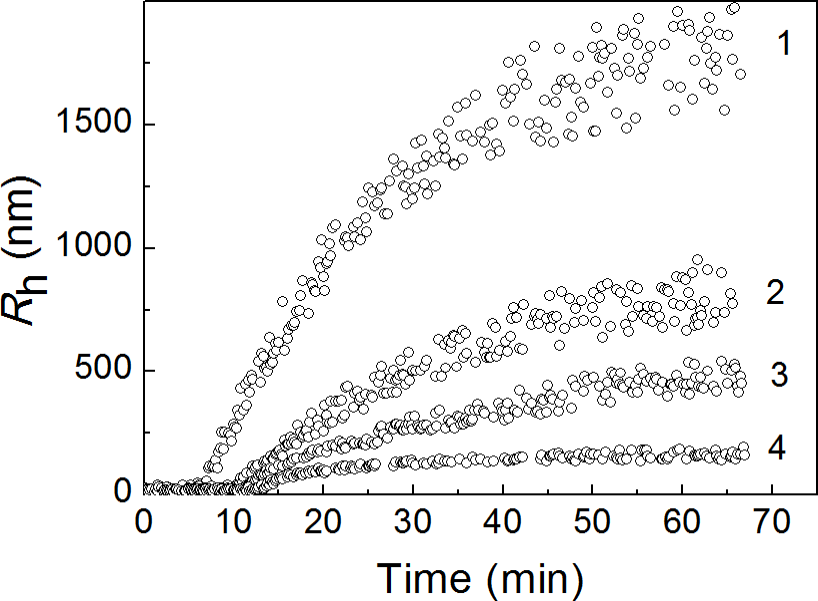
The dependences of the hydrodynamic radius (*R*_h_) of the particles formed upon addition of DTT to the solution of insulin (0.32 mg/mL) on time in the presence of α-crystallin. The concentrations of α-crystallin were as follows: 0.08 (1), 0.32 (2), 0.64 (3), and 0.80 (4) mg/mL.

Figure 7 shows the dependences of the hydrodynamic radius of the protein aggregates formed in the solution of insulin (0.32 mg/mL) on time after the addition of DTT in the presence of α-crystallin at concentrations of 0.08, 0.32, 0.64 and 0.8 mg/mL (curves 1, 2, 3 and 4, respectively). As can be seen from Figure 7, the higher the concentration of α-crystallin, the lower the size of the aggregates.

**Figure 8.**
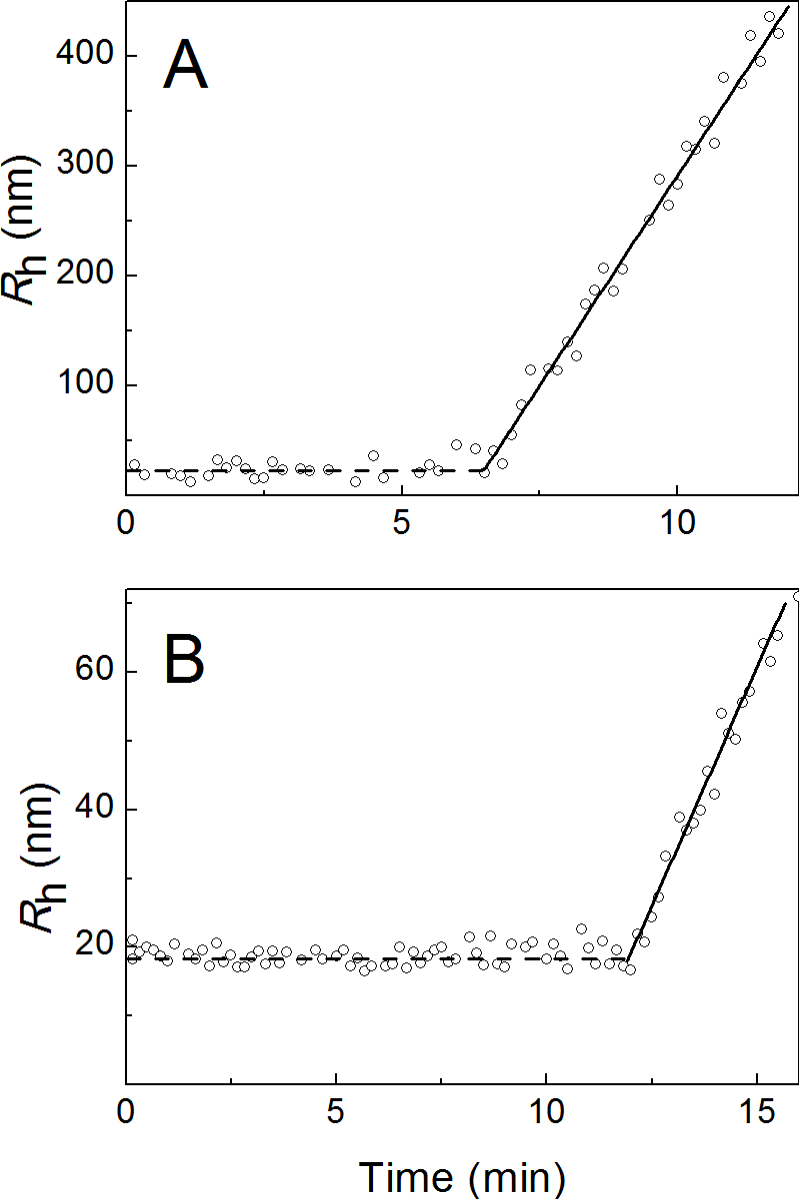
The analysis of the initial parts of the dependences of the hydrodynamic radius (*R*_h_) on time for aggregation of insulin (0.32 mg/mL) in the presence of α-crystallin. The concentrations of α-crystallin were 0.08 (**A**) and 0.80 (**B**) mg/mL. Solid lines were calculated from Equation (2).

The shape of the initial parts of the dependences of *R*_h_ on time is of special interest. As an example, Figure 8 shows the initial parts of such dependences in the presence of α-crystallin at the concentration of 0.08 mg/mL (Figure 8A) and 0.8 mg/mL (Figure 8B).

Immediately after the addition of DTT, the particles with *R*_h_ = 24.6 ± 1.2 nm (Figure 8A) and 18.4 ± 0.8 nm (Figure 8B) are registered in the system. The value of *R*_h_ remains constant over 6.5 and 11.8 min, respectively. When interpreting these results, we take into account that judging from the DLS measurements, the hydrodynamic radius of the α-crystallin preparation used in the present work was 13.2 ± 0.1 nm. One can assume that the particles with *R*_h_ = 24.6 and 18.4 nm are the complexes of α-crystallin with the insulin B chain. Let us designate the hydrodynamic radius of these complexes as *R*_h,complex_. At a definite point in time, the hydrodynamic radius of the primary particles begins to increase. The linear parts of the increase in the *R*_h_ value in time were treated using an Equation (5).

Parameters *K*_1_ and *t*_3_ are given in Table 2. The comparison of the values of *t*_2_ and *t*_3_ shows that the moment in time at which the light scattering intensity begins to increase (*t*_2_), coincides with the time at which the size of the α-crystallin–insulin B chain complexes begins to increase (*t*_3_). The rate of the increase in the *R*_h_ value in time (parameter *K*_1_) gradually decreases on increasing the α-crystallin concentration.

**Figure 9.**
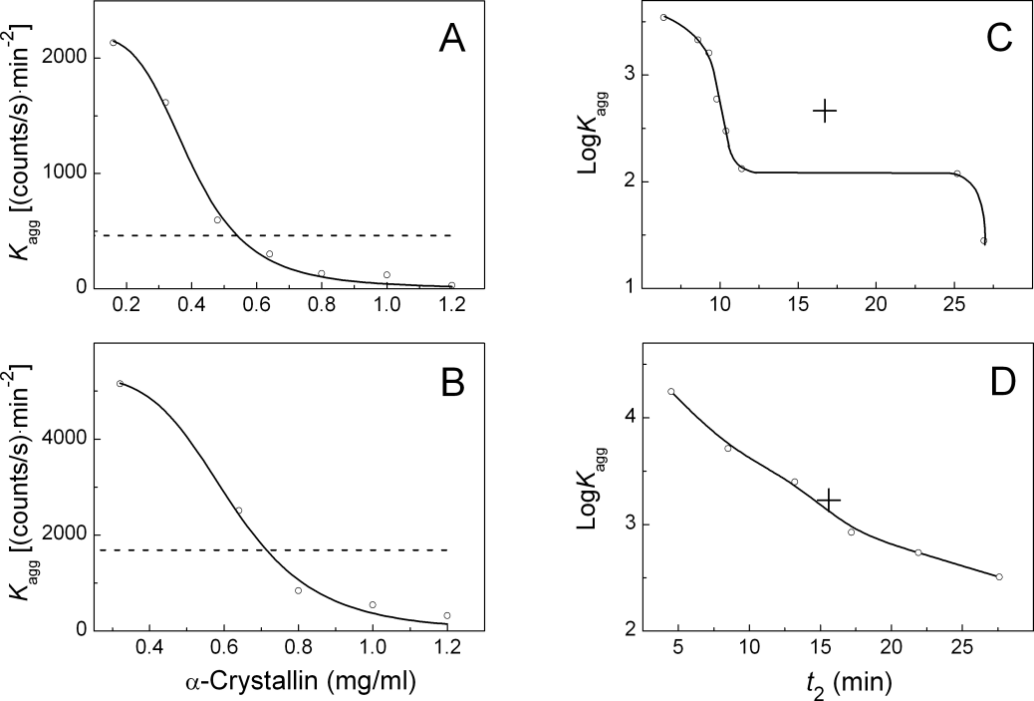
Analysis of parameters *K*_agg_ and *t*_2_ used for description of the dependences of the light scattering intensity on time for DTT-induced aggregation of insulin in the presence of various concentrations of α-crystallin. The dependences of parameter *K*_agg_ on the concentration of α-crystallin at the concentrations of insulin of: 0.32 (**A**) and 0.4 (**B**) mg/mL. The horizontal dotted lines correspond to the *K*_agg_ values obtained for insulin aggregation in the absence of α-crystallin ( $K_{\text{agg}}^{\text{control}}$). The solid curves were drawn from Equation (5). The relationships between Log *K*_agg_ and parameter *t*_2_ at the concentrations of insulin of: 0.32 (**C**) and 0.4 (**D**) mg/mL. Crosses correspond to insulin aggregation in the absence of α-crystallin.

Figure 9A and B demonstrates the dual effect of α-crystallin on the initial rate of insulin aggregation. The horizontal dotted lines in this figure correspond to the *K*_agg_ value measured in the absence of α-crystallin ( $K_{\text{agg}}^{\text{control}}$). If the values of *K*_agg_ are located above the control level, acceleration of insulin aggregation takes place, whereas the *K*_agg_ values below the control level correspond to suppression of aggregation. To determine α-crystallin concentration, at which the initial rate of aggregation coincides with the value of *K*_agg_ measured in the absence of α-crystallin, we used the Equation (6).

It should be noted that *K*_agg,0_ is the expected value of *K*_agg_, if the mechanism of insulin aggregation realized in the presence of α-crystallin remains intact at *C* → 0. Since the mechanisms of insulin aggregation which are operative in the presence or in the absence of α-crystallin are different, the *K*_agg,0_ value is not identical to $K_{\text{agg}}^{\text{control}}$. The following values of parameters *K*_agg,0_, *C*_0.5_ and *h* were found using this equation: *K*_agg,0_ = 2180 ± 40 (counts/s)·min^−2^, *C*_0.5_ = 0.40 ± 0.01 mg/mL and *h* = 4.3 ± 0.2 at the insulin concentration of 0.32 mg/mL, and *K*_agg,0_ = 5310 ± 160 (counts/s)·min^−2^, *C*_0.5_ = 0.62 ± 0.02 mg/mL and *h* = 5.4 ± 0.5 at the insulin concentration of 0.4 mg/mL.

With the knowledge of these parameters, we calculated the concentration of α-crystallin (C*), at which the *K*_agg_ value is numerically equal to $K_{\text{agg}}^{\text{control}}:C*=C_{0.5}{\left(K_{\text{agg},0}/K_{\text{agg}}^{\text{control}}-1\right)}^{1/h}$. The values of $K_{\text{agg}}^{\text{control}}$ are equal to 460 and 1690 (counts/s)·min^−2^ at the insulin concentration of 0.32 and 0.4 mg/mL, respectively. The corresponding values of C* were found to be 0.55 and 0.71 mg/mL, respectively. Insets in Figure 9C and D show the relationships between the parameters *K*_agg_ and *t*_2_ in the coordinates {log*K*_agg_; *t*_2_}. When the concentration of insulin was 0.4 mg/mL, the point with coordinates corresponding to the values of *K*_agg_ and *t*_2_, measured in the absence of α-crystallin (this point is designated by a cross), fall on the curve connecting the points corresponding to *K*_agg_ and *t*_2_ values measured in the presence of α-crystallin. This explains the fact that the effect of α-crystallin on aggregation of insulin is clearly visible when the concentration of α-crystallin was 0.4 mg/mL (Figure 5B). As for the dependence of log *K*_agg_ on *t*_2_ at the insulin concentration of 0.32 mg/mL, the point corresponding to the control values of *K*_agg_ and *t*_2_ lies outside the curve connecting the points corresponding to the *K*_agg_ and *t*_2_ values measured in the presence of α-crystallin. This results in the complicated influence of α-crystallin on insulin aggregation (Figure 5A).

**Figure 10.**
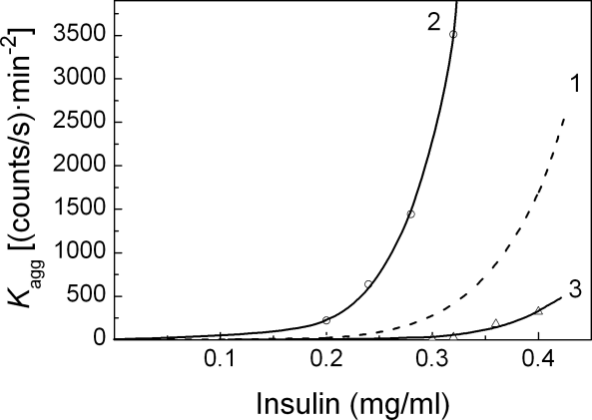
The effect of α-crystallin on the dependence of parameter *K*_agg_ on the concentration of insulin. The concentrations of α-crystallin were as following: 0 (1), 0.08 (2) and 1.20 (3) mg/mL. The points are the experimental data; the solid curves were calculated from Equation (4).

It was of interest to study the effect of α-crystallin on the shape of the dependences of the initial rate of aggregation characterized by parameter *K*_agg_ on the insulin concentration. Figure 10 shows these dependences obtained in the absence (curve 1) and in the presence of α-crystallin at the concentrations of 0.08 and 1.2 mg/mL (curves 2 and 3, respectively). First of all, the dependences of parameter *K*_agg_ on the insulin concentration presented in this figure demonstrate the accelerating effect of the relatively low concentrations of α-crystallin (curve 2) and the inhibiting action of rather high concentrations of α-crystallin (curve 3). Furthermore, the dependences under discussion obtained in the presence of α-crystallin, similar to the corresponding dependence obtained in the absence of α-crystallin, follow the power law presented by Equation (4). The value of parameter *n* at the α-crystallin concentration of 0.08 mg/mL (*n* = 7.8 ± 0.2) exceeds the corresponding value obtained in the absence of α-crystallin (*n* = 6.3 ± 0.2). When the concentration of α-crystallin was 1.2 mg/mL, the value of this parameter (*n* = 5.9 ± 0.2) was close to that for the dependence of parameter *K*_agg_ on the insulin concentration obtained in the absence of α-crystallin.

### 2.3. Fluorescence Studies of α-Crystallin Interaction with Insulin

The formation of the complex between two proteins was also confirmed by the tryptophan fluorescence measurements. Since α-crystallin, but not insulin, contains tryptophan residues, we used fluorescence spectroscopy to examine the tryptophan emission spectra of the individual α-crystallin and the chaperone within the complex α-crystallin–insulin. α-crystallin taken alone gives fluorescence spectra with emission maximum at 335 nm (data not shown). The results of the experiments show that insulin, in its original form, does not bind to α-crystallin. In the absence of DTT, the fluorescence intensity values are almost equal to those of the sum of the individual fluorescence emission spectra of insulin and α-crystallin (data not shown), implying that there was little, if any, interaction between the two proteins. Only the aggregation-prone reduced insulin was found to interact with α-crystallin. On reduction of disulfide bonds of insulin by DTT, an almost immediate increment on the dependences of the relative fluorescence intensity on time registered at 335 nm was observed. However, no shift in the emission maximum of intrinsic fluorescence was detected.

**Figure 11.**
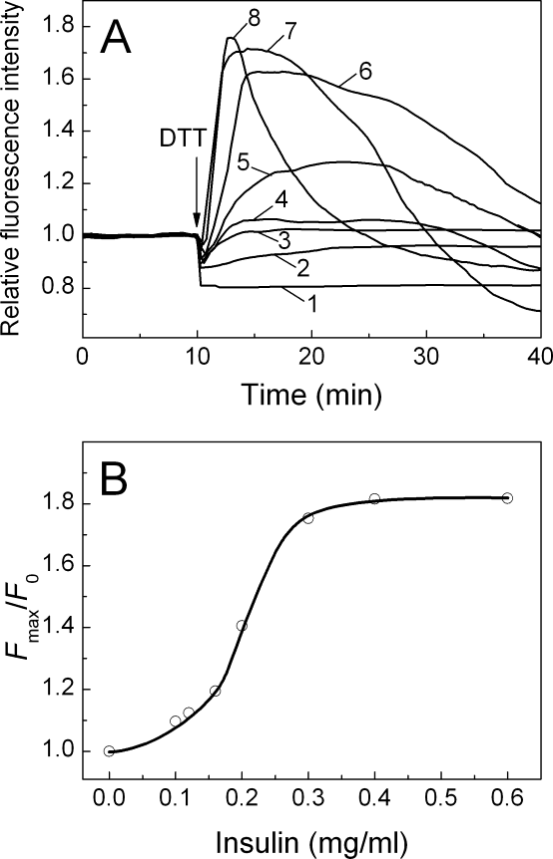
The interaction between insulin and α-crystallin monitored by tryptophan fluorescence. (**A**) The dependences of the relative fluorescence intensity on time of the solution containing α-crystallin at the concentration of 0.3 mg/mL (1) or the mixtures of α-crystallin (0.3 mg/mL) and insulin at the concentrations of 0.1 (2), 0.12 (3), 0.16 (4), 0.2 (5), 0.3 (6), 0.4 (7) and 0.6 (8) mg/mL. The arrow shows the moment of addition of DTT. The final concentration of DTT was 20 mM. The fluorescence was recorded at 25 °C in 50 mM sodium phosphate buffer, pH 7.0, containing 0.15 M NaCl upon excitation and emission at 297 and 335 nm, respectively; (**B**) Dependence of the increment of the fluorescence intensity (*F*_max_/*F*_0_) on the concentration of insulin. *F*_0_ is the minimum value of the fluorescence intensity reached immediately after the addition of insulin and *F*_max_ is the maximum value of the fluorescence intensity.

At the α-crystallin concentration of 0.3 mg/mL, the increment was shown to increase in a concentration-dependent manner in the presence of insulin at concentrations from 0.2 to 0.6 mg/mL (Figure 11A, curves 5–8, respectively) corresponding to the molar stoichiometric ratio (insulin:α-crystallin) less than 1:0.5. The increase in the intensity of tryptophan fluorescence of α-crystallin, under the conditions of the appearance of aggregation-prone reduced insulin, suggests that the α-crystallin–insulin complex is formed and that the tertiary structure of the complex is more compact than that of the individual chaperone [22].

At molar stoichiometric ratio (insulin:α-crystallin) exceeding the value of 1:0.6, at which α-crystallin caused suppression of insulin aggregation, almost no increment on the dependences of the relative fluorescence intensity on time was shown (Figure 11A, curves 2 and 3). Bovine α-crystallin possesses no disulphide bonds and therefore no change in its tryptophan emission spectrum could be observed upon addition of DTT; however, a quenching of tryptophan emission intensity by DTT was detected, the most pronounced effect being revealed in the absence of insulin (Figure 11A, curve 1).

The dependence of the relative increment of the fluorescence intensity (*F*_max_/*F*_0_) on the concentration of insulin is also shown (Figure 11B), where *F*_0_ is the minimum value of the fluorescence intensity reached immediately after the addition of insulin, and *F*_max_ is the maximum value of the fluorescence intensity. At the fixed concentration of α-crystallin (0.3 mg/mL), the increment amplitude gradually increased at a concentration of insulin in the range from 0.05 to 0.4 mg/mL; whereas at higher insulin concentrations, no further increase of the relative fluorescence intensity (*F*_max_/*F*_0_) was observed.

In our experiments, an increase in tryptophan fluorescence of α-crystallin in the presence of insulin after the addition of DTT is followed by a gradual decrease in the fluorescence intensity at further incubation (Figure 11A, curves 4–8). The moment in time at which the fluorescence intensity begins to decrease, correlates well with the kinetics of DTT-induced aggregation of insulin (data not shown). Thus, the diminishing of the fluorescence intensity at rather high values of time is due to the increase in turbidity of the solution.

## 3. Discussion

First of all, consider what information may be obtained from analysis of the dependences of the initial aggregation rate characterized by parameter *K*_agg_, which is calculated from the light scattering intensity *versus* time plots, on the concentration of the protein substrate. In [23], where the quadratic equation was proposed for the description of the dependence of the light scattering intensity on time, the dependence of parameter *K*_agg_ on the concentration of the protein substrate was analyzed for heat aggregation of glycogen phosphorylase *b* from rabbit skeletal muscle. The experimental data obtained by Kornilaev *et al.* [24] were used for the construction of the parameter *K*_agg_ *versus* glycogen phosphorylase *b* concentration plot. It was found that parameter *K*_agg_ was a linear function of the glycogen phosphorylase *b* concentration. This means that parameter *n* in Equation (4) is equal to unity and, consequently, the rate-limiting stage of the overall process of aggregation is the monomolecular stage of unfolding of the protein molecule. This conclusion agrees with the mechanism of thermal denaturation of glycogen phosphorylase *b*, established by Kurganov *et al.* [25]. The kinetic scheme of thermal denaturation of glycogen phosphorylase *b*, involves the stage of the conformational change of the dimeric molecule of the enzyme, followed by dissociation of dimer into monomers, and denaturation of labile monomeric forms.

When studying DTT-induced aggregation of α-lactalbumin [3], we showed that unfolding of the α-lactalbumin molecule proceeded immediately after the addition of DTT. Thus, the rate-limiting stage of the process of α-lactalbumin aggregation corresponds to the stage of the interaction of the unfolded protein molecules, namely the nucleation stage. The analysis of the dependence of parameter *K*_agg_ on the concentration of α-lactalbumin presented in the work [3] shows that parameter *n* calculated from Equation (4) is equal to 3.5 ± 0.2. The fact that the value of parameter *n* exceeds unity, is indicative of the involvement of several molecules of unfolded α-lactalbumin in the nucleation process. The results of the study of DTT-induced aggregation of insulin carried out in the present work, show that parameter *n* essentially exceeds unity (*n* ≥ 6). By this is meant that the rate-limiting stage of the process of DTT-induced aggregation of insulin, as in the case of DTT-induced aggregation of α-lactalbumin, is the stage of nucleation.

It is generally accepted that the appearance of lag phase on the kinetic curves of aggregation indicates that the initial stage of aggregation is the stage of nucleation [23,26–33]. The formation of a nucleus proceeds as a result of cooperative association of several molecules of the unfolded protein substrate. The study of the kinetics of protein aggregation using DLS showed that the nuclei could not be detected because of their rapid sticking to form large-sized assemblies. It has beenthese large-sized assemblies that are registered as start aggregates by DLS [3,34].

The estimation of the size of the protein aggregates formed in the course of DTT-induced aggregation of insulin, showed that the initially registered aggregates are the start aggregates with a hydrodynamic radius of 94 ± 7 nm. It is of interest that the size of the start aggregates remains unchangeable on variation of the insulin concentration. This fact allows us to draw an analogy between the formation of the start aggregates and micelle formation. In the latter case, the micelles of a definite size are formed when the critical micelle concentration is achieved. Such an analogy offers an explanation for the fact that the formation of start aggregates proceeds according to the all-or-none principle.

The analyses of the dependences of the hydrodynamic radius of the protein aggregates on time for DTT-induced aggregation of insulin showed that the *R*_h_ value did not approach the limiting value even after prolonged incubation. The increase in the *R*_h_ value over time follows the power law (*R*_h_ ∼ *t*^1/1.8^ = *t*^0.56^). The *d*_f_ value calculated from Equation (3) was found to be close to 1.8. This fact suggests that the growth of the start aggregate proceeds as a result of sticking of the start aggregates, rather than due to the attachment of the unfolded insulin molecule to a nucleus, as is postulated in the nucleation-dependent models of aggregation.

When studying the effect of α-crystallin on the kinetics of DTT-induced aggregation of insulin, we demonstrated that relatively high concentrations of α-crystallin suppressed insulin aggregation. This result agrees with the traditional point of view that the main function of α-crystallin, as a representative of the family of small heat shock proteins, is a suppression of aggregation of the unfolded protein molecules while maintaining non-native proteins in a refolding competent conformation [1]. However, detailed studies of the effect of low concentrations of α-crystallin on the kinetics of DTT-induced aggregation of protein substrates have not yet been conducted.

**Scheme 1.**
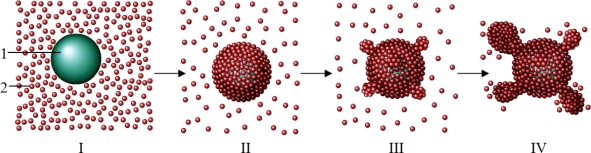
Schematic representation of DTT-induced aggregation of insulin in the presence of relatively low concentrations of α-crystallin causing acceleration of the aggregation process (1 is the α-crystallin particle and 2 is the unfolded B chain of insulin).

The results obtained in the present work clearly demonstrate that relatively low concentrations of α-crystallin accelerate DTT-induced aggregation of insulin. Such an effect of α-crystallin may be provoked by the stimulation of the nucleation stage as a result of binding of the unfolded insulin B chain to the surface of the α-crystallin particle. To clarify this idea, Scheme 1 illustrates DTT-induced aggregation of insulin in the presence of low concentrations of α-crystallin. State I in this scheme is a demonstration of the components initially present in the system, namely α-crystallin particles (1) and unfolded B chain of insulin [2]. The high values of (unfolded B chain of insulin):(α-crystallin) ratio favor filling of the surface of the α-crystallin particle by adsorbed B chain of insulin (state II). The high local concentrations of unfolded protein molecules on the surface of the α-crystallin particle facilitates the stage of nucleation, which should be called the heterogeneous nucleation because it proceeds on the surface of the rather large particle [35] (state III). In the scheme, nuclei are depicted as “nodules” on the surface of the α-crystallin particle. The formed nuclei serve as the points of growth of the aggregates including the unfolded B chains of insulin (state IV). Owing to concentrating the unfolded protein molecules on the surface of the α-crystallin particle, the heterogeneous nucleation in the presence of α-crystallin proceeds more effectively than the homogenous nucleation of the unfolded protein in the absence of α-crystallin. This explains the acceleration of DTT-induced aggregation of insulin in the presence of relatively low concentrations of α-crystallin.

**Scheme 2.**
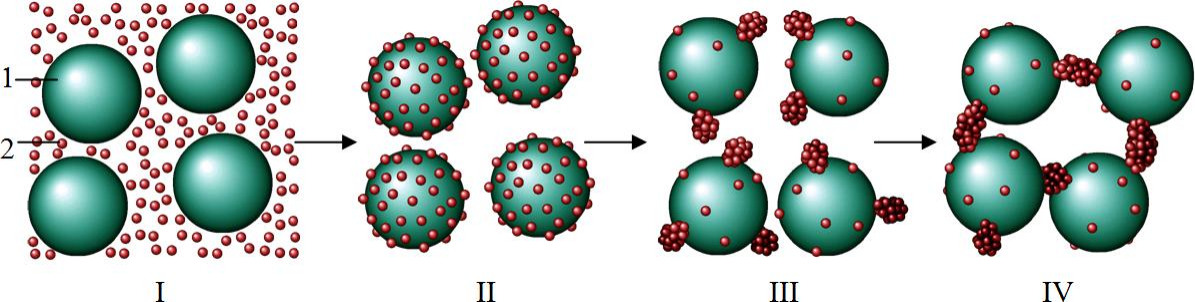
Schematic representation of DTT-induced aggregation of insulin in the presence of relatively high concentrations of α-crystallin, causing suppression of the aggregation process (1 is the α-crystallin particle and 2 is the unfolded B chain of insulin).

Consider the region of the α-crystallin concentrations where the chaperone acts as a suppressor of insulin aggregation (Scheme 2). At rather low values of (unfolded B chain of insulin):(α-crystallin) ratio interaction of chaperone and the protein substrate results in the formation of the complexes which are not prone to aggregation (state II in Scheme 2). However, after a certain period of time aggregation of these complexes occurs (see, for example, Figure 8). The initial stage of aggregation is the formation of nuclei on the surface of the α-crystallin particle (state III in Scheme 2). At α-crystallin concentrations under discussion, a relatively low density of adsorbed insulin B chains on the surface of the α-crystallin particle hampers the formation of nuclei. The higher the α-crystallin concentration, the higher the duration of the lag period (*i.e.*, the higher the *t*_3_ value; see Table 2). The nuclei formed on the surface of the α-crystallin particle, serve as the sticky sites providing aggregation of the unfolded protein–α-crystallin complexes (state IV in Scheme 2).

One can assume that in the region of α-crystallin concentrations where the rates of aggregation measured in the absence or in the presence of α-crystallin are comparable, both mechanisms of aggregation presented in Schemes 1 and 2 are realized.

Thus, the results of the present work suggest that heterogeneous nucleation plays the key role in the realization of the mechanisms of DTT-induced aggregation of insulin proceeding in the presence of a molecular chaperone, α-crystallin. At rather high concentrations of α-crystallin, it has been solely heterogeneous nucleation with the participation of unfolded protein molecules rather sparsely distributed on the surface of the α-crystallin particle, which determines the delay in the aggregation process and relatively low rate of aggregation. At rather low concentrations of α-crystallin, drawing together of the unfolded protein molecules on the surface of the α-crystallin particle, favors heterogeneous nucleation and provides the higher rate of aggregation.

The latter case may have substantial biological relevance. In response to a cellular stress, which generates a large amount of misfolded or partially folded proteins (hyperthermia, overexpression of insoluble or mutant proteins, *etc.*), cells are protected from accumulating protein aggregates by mechanisms involving the suppression of aggregate formation by molecular chaperones and degradation of pathogenic proteins by the ubiquitin-proteasome pathway. At the initial stages of the protein aggregation processes induced by different kinds of stress, the level of Hsp concentrations in a cell may be rather low. Acceleration of protein aggregation triggered by Hsps resulting in the formation of large-sized aggregates may have positive consequences. We suggest that rapidly generated amorphous protein aggregates on the surface of Hsp particles, can act as a means to concentrate the unfolded protein molecules within the cytosol of a cell in a manner analogous to the role of aggresomes [36,37]. An aggresome is a proteinaceous inclusion body that forms in response to a stress, when cellular chaperoning or degradation machinery is impaired, leading to a protective biological effect as a result of accumulation of abnormal or damaged proteins within the inclusion bodies.

In the present work, an accelerated targeting of the stress-induced unfolded protein substrate to the α-crystallin particles is assumed to be a protective response, resembling that of an aggresome, sequestering wide distribution of potentially toxic aggregates.

## 4. Experimental Section

### 4.1. Materials

Human recombinant insulin and DTT were purchased from Sigma. All other chemicals were of reagent grade. All solutions for the experiments were prepared using deionized water obtained with Easy-Pure II RF system (Barnstead, U.S.).

### 4.2. Isolation and Purification of α-Crystallin

Purification of α-crystallin from freshly excised lenses of two-year-old steers was performed according to the procedure described earlier [38]. The α-crystallin concentration was determined from the absorbance at 280 nm using the extinction coefficient $A_{208}^{1\%}$ of 8.5 [39].

### 4.3. DTT-Induced Aggregation of Insulin

The kinetics of insulin aggregation was registered in 50 mM Na-phosphate buffer, pH 7.0, containing 0.15 M NaCl. Insulin was dissolved in 20 mM NaOH and then in sodium phosphate buffer to a concentration of 1 mg/mL. An aliquot of this solution was added to the buffer in the cell. Reduction of insulin (0.2–0.4 mg/mL) was initiated by adding DTT to 0.5 mL of the sample to a final concentration of 20 mM. To study the effect of α-crystallin on insulin aggregation, aliquots of α-crystallin solutions were added into the cell simultaneously with insulin. The experiments were performed at room temperature (25 °C).

### 4.4. Dynamic Light Scattering Studies

DLS measurements were performed by a commercial instrument Photocor Complex (Photocor Instruments Inc., U.S.; www.photocor.com) as described in our previous works where DLS was used for the study of the kinetics of thermal and DTT-induced aggregation of proteins [3,40]. A He-Ne laser (Coherent, U.S., Model 31–2082, 632.8 nm, 10 mW) was used as a light source. The temperature of sample cell was controlled by the proportional integral derivative (PID) temperature controller to within ±0.1 °C. The quasi-cross correlation photon counting system with two photomultiplier tubes was used to increase the accuracy of particle sizing in the range from 1.0 nm to 5.0 μm. DLS data have been accumulated and analyzed with multifunctional real-time correlator Photocor-FC. DynaLS software (Alango, Israel) was used for polydispersity analysis of the DLS data. The diffusion coefficient *D* of the particles is directly related to the decay rate τ_c_ of the time-dependent correlation function for the light-scattering intensity fluctuations: *D* = 1/2τ_c_*k*^2^. In this equation, *k* is the wavenumber of the scattered light, *k* = (4τ*n*/λ)sin(θ/2), where *n* is the refractive index of the solvent, λ is the wavelength of the incident light in vacuum and θ is the scattering angle. The mean hydrodynamic radius of the particles (*R*_h_) can then be calculated according to the Stokes-Einstein Equation: *D* = *k*_B_*T*/6τη*R*_h_, where *k*_B_ is Boltzmann’s constant, *T* is the absolute temperature and η is the shear viscosity of the solvent.

To analyze the time-course of the increase in the light scattering intensity (*I*) accompanying aggregation of insulin, we used the approaches elaborated by us previously [23]. The following designations were used. The time of the appearance of the start aggregates was designated as *t*_1_, and the designation *t*_2_ was used for the duration of the lag period on the dependences of the light scattering intensity on time. To calculate parameter *t*_2_, in the present work we used the empiric equation:

(1) $$I=K_{\text{agg}}{\left(t-t_{2}\right)}^{2}$$

where *K*_agg_ is a constant with the dimension of (counts/s)·min^−2^. Parameter *K*_agg_ characterizes the initial rate of aggregation.

It should be noted that in some of our previous works (see, for example [3,41,42]) we used Equation (2) in the form: *I* = *K*_LS_(*t* − *t*_2_)^2^. The constant *K*_LS_ in this equation has the following dimension: (counts/s)^1/2^·min^−1^. DLS allows monitoring the course of the protein aggregation by the increase in the hydrodynamic radius (*R*_h_) of the growing particles. In this case, the rate of aggregation may be characterized by the value of 1/*t*_2R_, where *t*_2R_ is the time interval, over which the *R*_h_ value is doubled. Since the dimension of the 1/*t*_2R_ value is min min^−1^, we can directly compare parameter *K*_LS_, which is calculated from the dependences of the light scattering intensity on time, with parameter 1/*t*_2R_, which is calculated from the dependences of *R*_h_ on time. It is evident that parameters *K*_agg_ and *K*_LS_ are connected by the following relationship: *K*_agg_ *=* (*K*_LS_)^2^.

To analyze the dependences of the hydrodynamic radius (*R*_h_) of the protein aggregates on time for DTT-induced aggregation of insulin, we used the approaches applied in the cases of thermal aggregation of proteins [40,43]. The following equation may be used for the description of the initial linear parts of the dependences of *R*_h_ on time:

(2) $$R_{\text{h}}=R_{\text{h},0}+K_{1}(t-t_{3})$$

where *R*_h,0_ is the hydrodynamic radius of the start aggregates, *t*_3_ is the point in time at which the hydrodynamic radius of the start aggregates begins to increase and *K*_1_ is a constant.

When studying the kinetics of thermal aggregation of proteins, we observed that the typical dependences of the hydrodynamic radius (*R*_h_) of the protein aggregates on time were as follows. The initial parts of the dependences of *R*_h_ on time were linear. Above the definite point in time designated as t* the dependences of *R*_h_ on time followed the power law. The following equation may be used for the description of these parts of the dependences of *R*_h_ on time:

(3) $$R_{\text{h}}=R_{\text{h}}^{*}{\left[1+K_{2}(t-t*)\right]}^{1/d_{f}}$$

where $R_{h}^{*}$ is the *R*_h_ value at *t* = t*, *K*_2_ is a constant and *d*_f_ is the fractal dimension of the aggregates. The *d*_f_ value was found to be close to 1.8. This value of *d*_f_ is typical of the regime of diffusion-limited cluster-cluster aggregation, wherein each collision of the interacting particles results in their sticking [44–46].

To analyze the dependence of parameter *K*_agg_ on the concentration of insulin, we used the equation:

(4) $$K_{\text{agg}}=B{\left[P\right]}_{0}^{n}$$

where [P]_0_ is the initial concentration of the protein, *n* is the order of aggregation with respect to protein and *B* is a constant.

The linear parts of the increase in the *R*_h_ value of the complexes of α-crystallin with the insulin B chain (designated as *R*_h,complex_) in time (Figure 8) were treated using an equivalent equation to Equation (2):

(5) $$R_{\text{h}}=R_{\text{h},\text{complex}}+K_{1}(t-t_{3})$$

Parameters *K*_1_ and *t*_3_ are given in Table 2.

To determine α-crystallin concentration, at which the initial rate of aggregation coincides with the value of *K*_agg_ measured in the absence of α-crystallin, we used the following empiric equation:

(6) $$K_{\text{agg}}=\frac{K_{\text{agg},0}}{1+{\left(C/C_{0.5}\right)}^{h}}$$

where *C* is the α-crystallin concentration, *K*_agg,0_ is the limiting value of the function *K*_agg_(*C*) at *C* → 0, *C*_0.5_ is the α-crystallin concentration, at which *K*_agg_/*K*_agg,0_ = 0.5, and *h* is a constant (the Hill coefficient).

### 4.5. Fluorescence Spectroscopy

The tryptophan fluorescence measurements were performed using a Cary Eclipse fluorescence spectrophotometer (Varian, Inc.) with a temperature-controlled cuvette holder. Samples were excited at 297 nm and fluorescence emission spectra were recorded from 300 to 400 nm. The excitation and emission slit widths were set at 2.5 nm. Before measurement, protein samples (α-crystallin or insulin) were preincubated for 10 min at 25 °C in 50 mM sodium phosphate buffer, pH 7.0, containing 0.15 M NaCl. The interaction between two proteins was initiated by addition of DTT to a final concentration of 20 mM. The incubation mixtures were then subjected to the fluorescence measurements in a quartz cuvette with a 1 cm path length. In these experiments the time of sample exposition to illumination was minimized in order to avoid the UV-induced spectral effects as a result of reduction of S–S bonds and subsequent structural rearrangements in the protein molecule.

The dependences of the relative fluorescence intensity on time of the solutions containing α-crystallin (0.3 mg/mL), in the absence or presence of various concentrations of insulin, in the range from 0.1 to 0.6 mg/mL, were recorded immediately after addition of DTT, upon excitation and emission at 297 and 335 nm, respectively. Each spectrum was the average of 5 scans.

## 5. Conclusions

In the present study, we demonstrate that low concentrations of α-crystallin, a member of the small heat shock protein (Hsp) family, dramatically accelerate the dithiothreitol-induced aggregation of model protein substrate, human recombinant insulin; whereas high concentrations of α-crystallin suppress insulin aggregation. The mechanisms underlying the dual effect of α-crystallin have been proposed. It is assumed that heterogeneous nucleation occurring on the surface of the α-crystallin particle plays the key role in the paradoxical acceleration of insulin aggregation by α-crystallin that may provide an alternative biologically significant pathway of the aggregation process. We suggest that rapidly generated amorphous protein aggregates on the Hsp particles, can act as a means to concentrate the unfolded protein molecules within the cytosol of a cell in a manner analogous to the role of aggresomes. An accelerated targeting of the stress-induced unfolded protein substrate to the Hsp particles is assumed to be a protective response, resembling that of an aggresome, sequestering wide distribution of potentially toxic aggregates.

## Figures and Tables

**Table 1.** Parameters of DTT-induced aggregation of insulin at 25 °C in 50 mM sodium phosphate buffer, pH 7.0, containing 0.15 M NaCl.

**[Insulin] (mg/mL)**	***t*_1_ (min)**	***t*_2_ (min)**	***K*_agg_, (counts/s min^−2^)**	***R*_h,0_ (nm)**	***t*_3_ (min)**	***K*_1_ (nm/min)**	t* (min)	***d*_f_**
0.20	18.5	18.4 ± 0.5	8.4 ± 0.6	84 ± 3	31.3 ± 0.4	11.9 ± 0.5	47	1.80 ± 0.01
0.24	18.3	18.3 ± 0.5	25 ± 2	81 ± 2	29.0 ± 0.3	13.6 ± 0.6	42	1.80 ± 0.01
0.28	17.7	17.5 ± 0.2	144 ± 3	101 ± 3	26.2 ± 0.3	20.3 ± 0.6	38	1.80 ± 0.01
0.32	17.2	16.7 ± 0.2	460 ± 10	100 ± 2	19.3 ± 0.2	26.6 ± 0.9	28	1.80 ± 0.01
0.36	16.8	16.2 ± 0.2	1270 ± 20	100 ± 2	18.1 ± 0.1	31.0 ± 0.8	24	1.79 ± 0.01
0.40	16.2	15.6 ± 0.2	1690 ± 30	99 ± 3	17.7 ± 0.2	60 ± 2	22	1.79 ± 0.01

*t*_1_ is the time of appearance of the start aggregates; *t*_2_ (the duration of the lag period of the dependences of the light scattering intensity on time) and *K*_agg_ (parameter characterizing the initial rate of aggregation) were calculated from Equation (1) (see Experimental Section); *R*_h,0_ is the average value of the hydrodynamic radius of the start aggregates; *t*_3_ is the point in time, at which the hydrodynamic radius of the start aggregates begins to increase; *K*_1_ is a constant calculated from Equation (2); t* is the point in time above which the dependence of *R*_h_ on time follows the power law represented by Equation (3); *d*_f_ is the fractal dimension of the aggregates.

**Table 2.** Parameters of DTT-induced aggregation of insulin in the presence of various concentrations of α-crystallin.

**[α-Crystallin] (mg/mL)**	***t*_2_ (min)**	***K*_agg_ (counts/s min^−2^)**		***R*_h,complex_ (nm)**	***t*_3_ (min)**	***K*_1_ (nm/min)**	
Concentration of insulin 0.32 mg/mL							
0.00	16.7 ± 0.1	460 ± 10					
0.08	6.4 ± 0.1	3460 ± 50		24.6 ± 1.2	6.5 ± 0.2	76.0 ± 5.0	
0.16	8.6 ± 0.1	2130 ± 50		24.6 ± 1.1	9.1 ± 0.3	56.0 ± 4.0	
0.32	9.3 ± 0.1	1620 ± 50		20.6 ± 0.8	10.0 ± 0.1	35.0 ± 2.0	
0.48	9.8 ± 0.2	600 ± 20		22.3 ± 1.2	10.4 ± 0.1	23.5 ± 1.9	
0.64	10.4 ± 0.1	300 ± 6		21.0 ± 0.8	11.1 ± 0.1	18.4 ± 0.8	
0.80	11.4 ± 0.1	132 ± 3		18.4 ± 0.8	11.8 ± 0.2	13.0 ± 0.5	
1.00	17.2 ± 0.3	119 ± 4		19.5 ± 0.8	17.7 ± 1.1	9.0 ± 0.5	
1.20	18.9 ± 0.5	28 ± 3		18.9 ± 0.5	19.4 ± 1.4	5.6 ± 0.5	
Concentration of insulin 0.4 mg/mL							
0.00	15.6 ± 0.1		1690 ± 30				
0.08	4.5 ± 0.1		17600 ± 350	22.6 ± 1.2	5.4 ± 0.1		82 ± 6
0.32	8.5 ± 0.1		5100 ± 100	21.8 ± 0.8	9.1 ± 0.2		71 ± 5
0.64	13.2 ± 0.1		2500 ± 100	23.1 ± 1.1	13.8 ± 0.5		63 ± 2
0.80	17.2 ± 0.1		840 ± 10	21.6 ± 0.8	17.9 ± 1.1		48.4 ± 1.8
1.00	21.9 ± 0.1		540 ± 10	21.2 ± 1.0	22.3 ± 1.2		23.0 ± 1.0
1.20	27.6 ± 0.1		321 ± 8	21.4 ± 0.9	27.9 ± 1.1		11.0 ± 0.8

*t*_2_ (the duration of the lag period of the dependences of the light scattering intensity on time) and *K*_agg_ (parameter characterizing the initial rate of aggregation) were calculated from Equation (1); *R*_h,complex_ is the hydrodynamic radius of the complex of α-crystallin with the insulin B chain; *t*_3_ is the point in time at which the *R*_h_ value begins to increase; *K*_1_ is a constant calculated from Equation (2).
